# A Rare Case of Leiomyosarcoma Next to the Iliac Crest

**DOI:** 10.7759/cureus.68970

**Published:** 2024-09-09

**Authors:** Achraf Tebbaa El Hassali, Mohammed Barrached, Adnane Lachkar, Najib Abdeljaouad, Hicham Yacoubi

**Affiliations:** 1 Orthopedics and Traumatology, Mohammed VI University Hospital, Faculty of Medicine and Pharmacy of Oujda, Mohamed I University, Oujda, MAR

**Keywords:** iliac crest, leiomyosarcoma, mass, muscle, tumor

## Abstract

Leiomyosarcoma is an aggressive soft tissue cancer frequently seen in the female gastrointestinal and genital tracts due to the preponderance of smooth muscle. We report the case of a patient with leiomyosarcoma next to the iliac crest, discussing this rare location with data from the literature.

## Introduction

Leiomyosarcoma is a malignant, aggressive soft tissue tumor that develops in smooth muscle tissue. This tumor can appear almost anywhere in the body, but most commonly begins in the female gastrointestinal and genital tracts due to the preponderance of smooth muscle at these sites [1]. The localization of the limbs is exceptional. There is no predominance linked to sex or age for extra-genital and extra-digestive locations. Currently, there is no specific cause that explains the occurrence of this sarcoma. The diagnosis is made by a biopsy, but leiomyosarcoma can also be discovered accidentally [2]. The prognosis varies depending on the location and size of the tumor.

We report the case of a patient with leiomyosarcoma next to the iliac crest, discussing this rare location with data from the recent scientific literature.

## Case presentation

We report the case of a 41-year-old patient, a mechanic by profession, married with two children. He had no notable personal or family pathological or substance use history. The patient consulted for the progressive appearance of a mass next to the right iliac crest two years before. The mass was painless and gradually increasing in size and evolving in a context of apyrexia and deterioration of general condition with significant asthenia and significant weight loss amounting to 20 kg in three months.

The clinical examination found a conscious patient, well-oriented in time and space. He was hemodynamically and respiratory stable: blood pressure at 135/76 mmHg, heart rate at 90 beats per minute, respiratory rate at 24 cycles per minute. The temperature was 37°C and the blood sugar level was 80 mg/dl.

**Figure 1:**
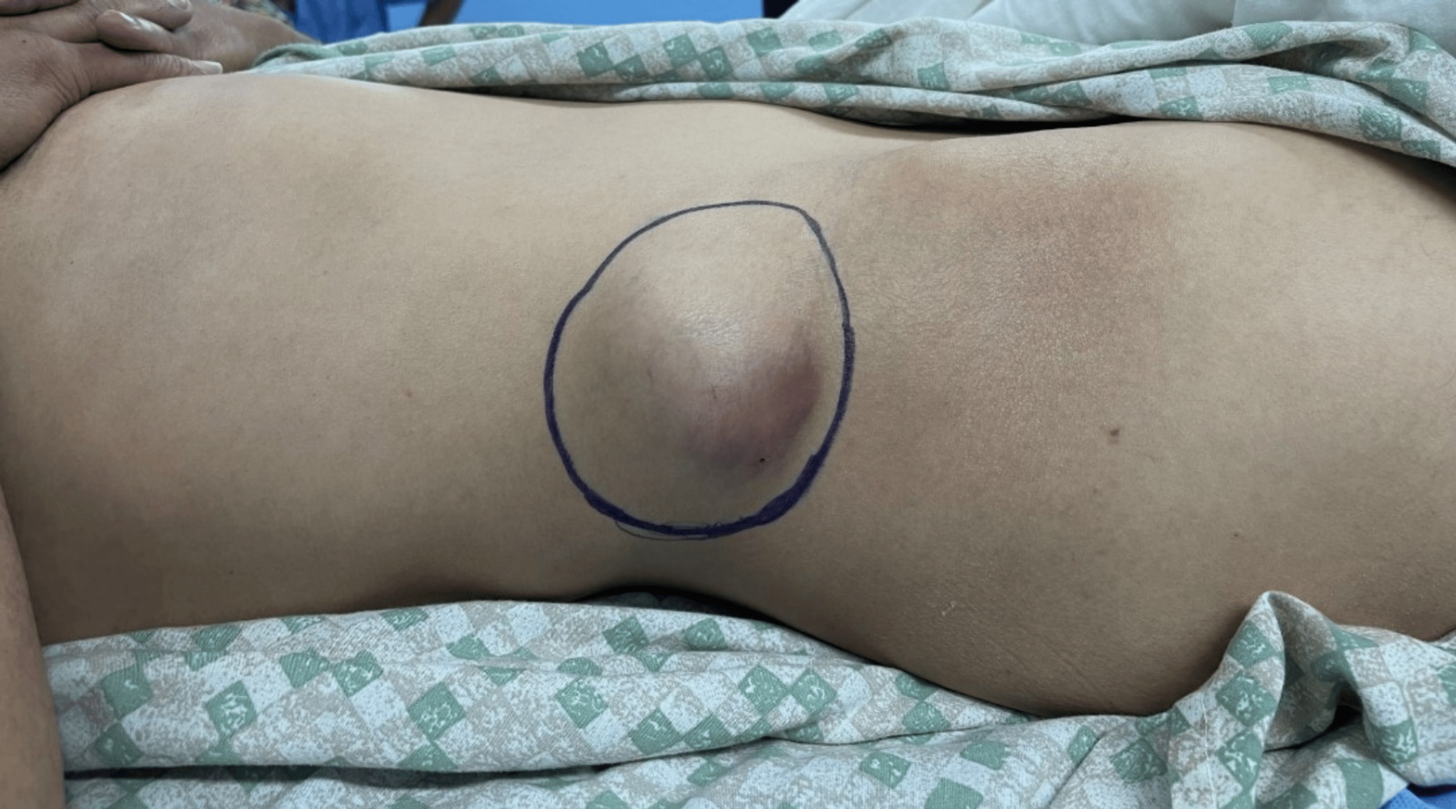
Lateral clinical image showing the mass at the level of the right iliac crest

Inspection found a rounded mass, well-defined next to the right iliac crest, approximately 7 cm in diameter without signs of inflammation or cutaneous fistulization. On palpation, the mass was hard and warm, adherent to the superficial and deep planes, and immobile. The lymph node areas were free and the vascular and nervous examination were without abnormality. The range of motion of the right hip was preserved (Figure 1).

The patient underwent a standard X-ray of the pelvis which did not show any abnormalities, supplemented by a computed tomography (CT) of the pelvis showing a soft tissue mass next to the right iliac crest of tissue density enhanced after injection of the contrast product, well limited and without adjacent bone lysis.

**Figure 2:**
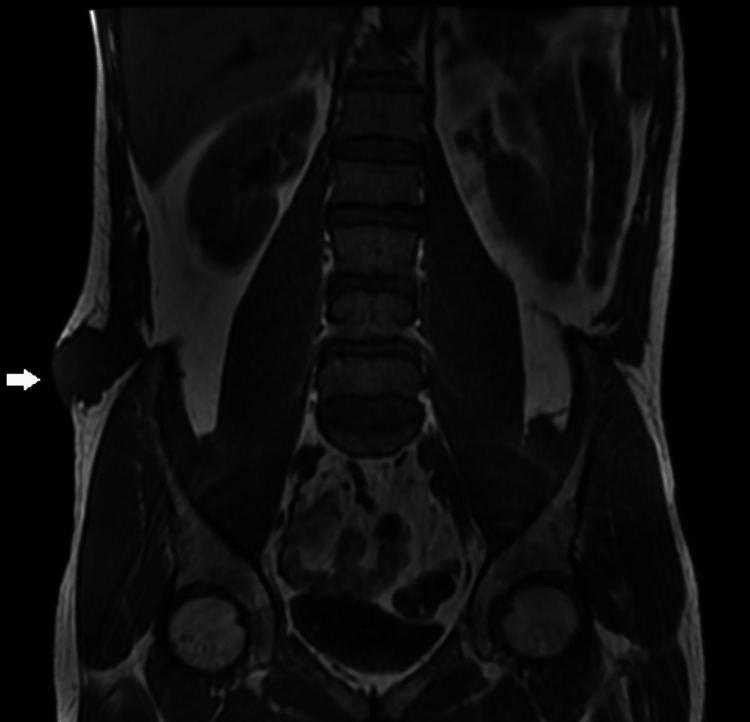
MRI image of a frontal section of the pelvis showing the tumor

The MRI showed a tumor mass of the soft anterolateral muscular parts of the right flank, well limited with regular contours, in T1 isosignal, T2 hypersignal, heterogeneous, containing fluid areas of necrosis, in diffusion hypersignal, enhancing heterogeneously after injection of contrast product. This mass appears to be of muscular origin at the level of the right external oblique muscle. It comes into intimate contact with the internal oblique muscle without a dividing line. It is vascularized by an arterial structure coming from the right lumbar artery opposite D12-L1. It projects opposite the antero-superior iliac spine with a separating fatty border. Outside, it infiltrates and deforms the skin (Figure 2).

A cerebro-thoraco-abdominal CT did not reveal any distant secondary localization. The complete biological assessment was without abnormalities.

An ultrasound-guided percutaneous biopsy was performed with histological and immunohistochemistry studies which returned in favor of a malignant spindle cell tumor process. A PET scan performed did not show a secondary location.

**Figure 3:**
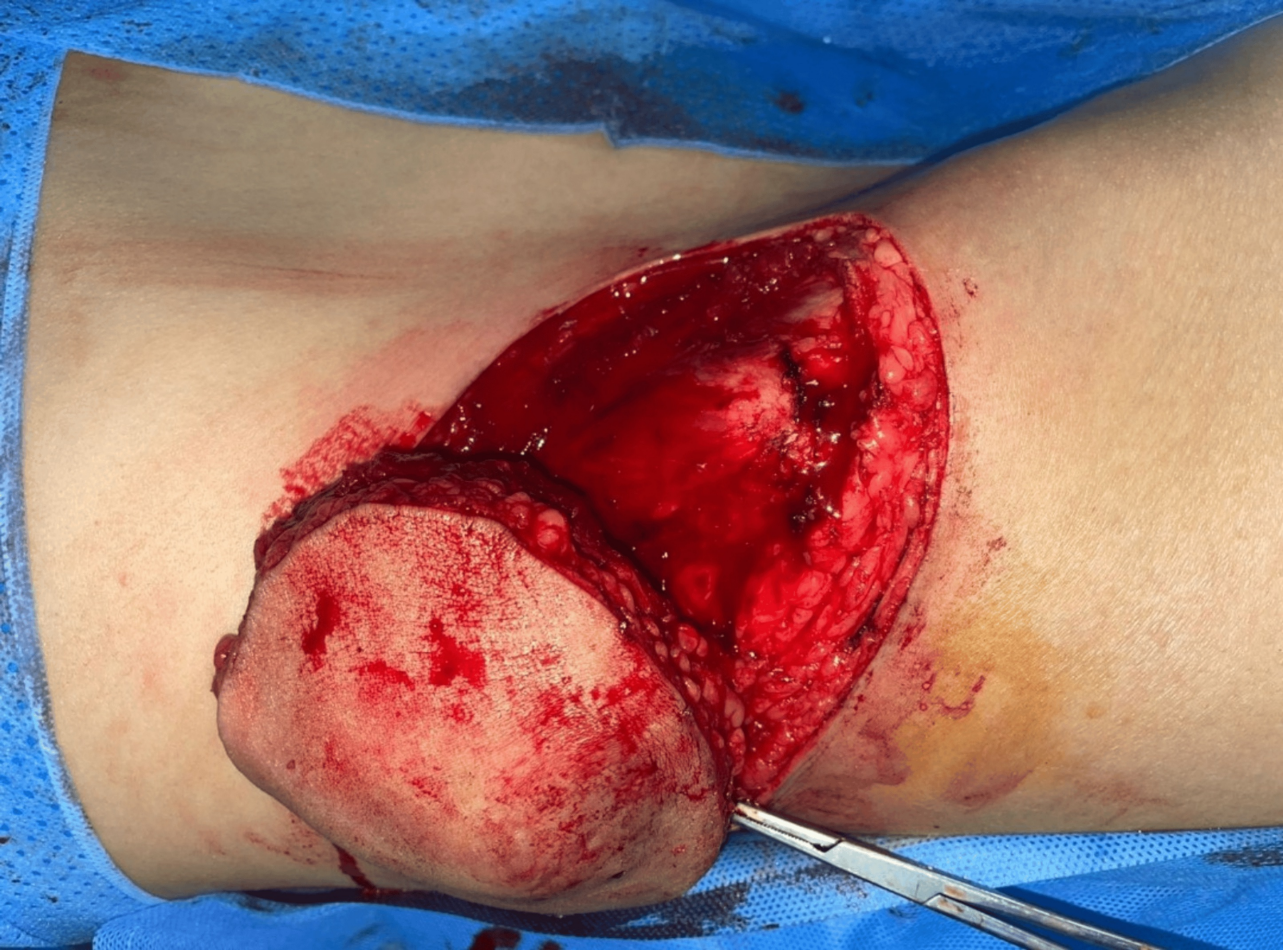
Operative image showing the tumor resection

**Figure 4:**
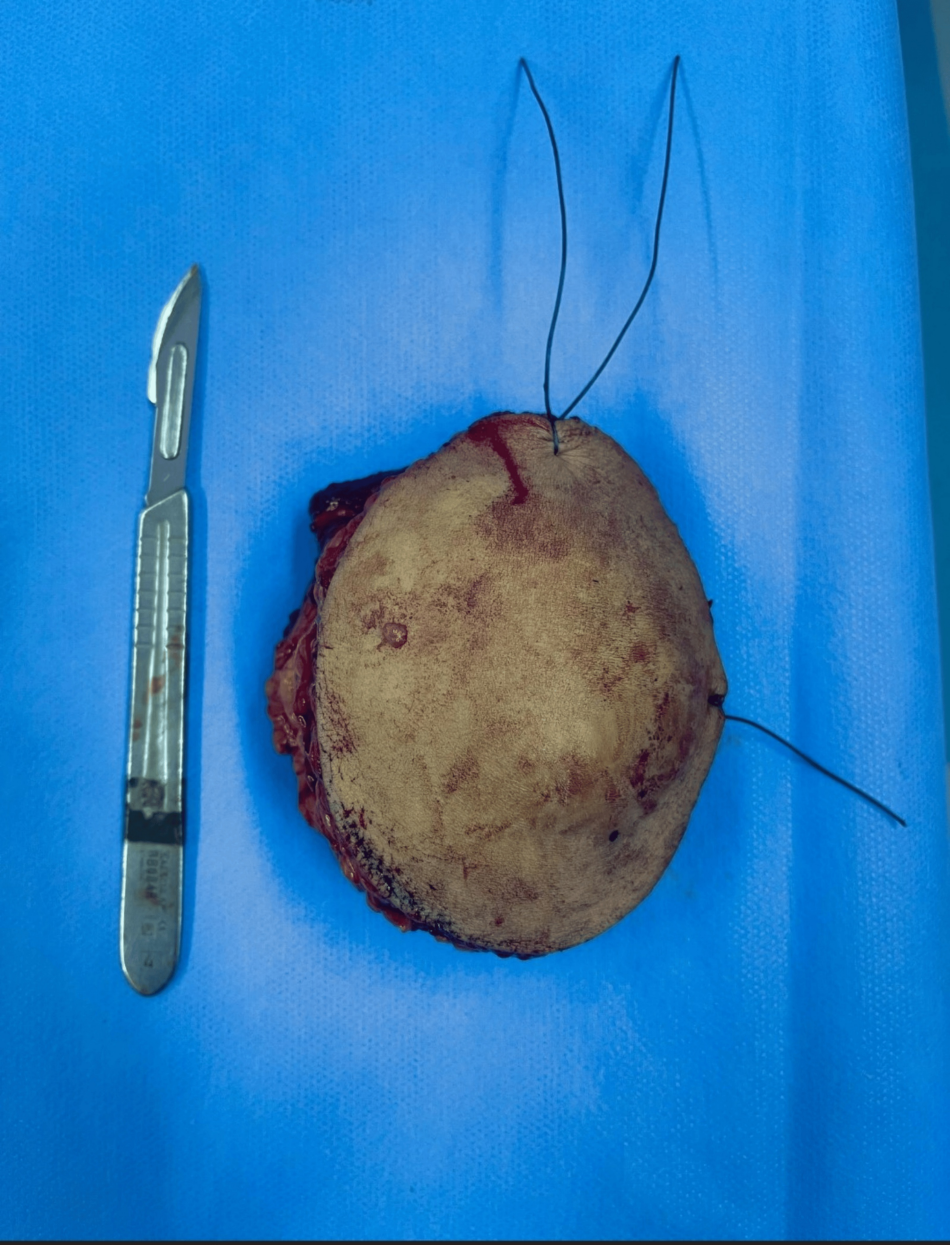
Operative image showing the tumor after resection

**Figure 5:**
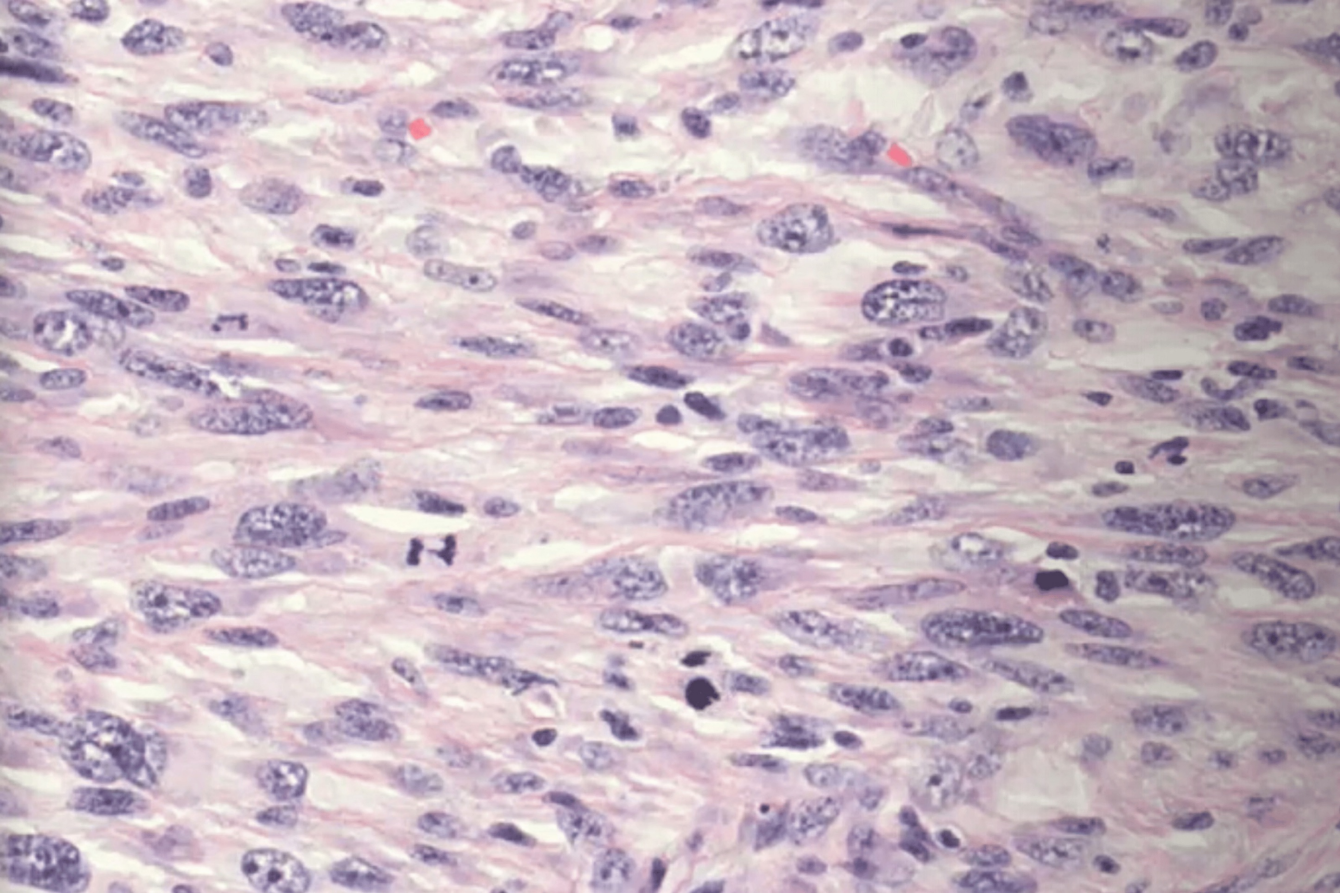
Photomicrograph of the lesion shows a spindle cell proliferation arranged in intersecting fascicles Tumor cells exhibit hyperchromatic and atypical nuclei (HES, x400)

**Figure 6:**
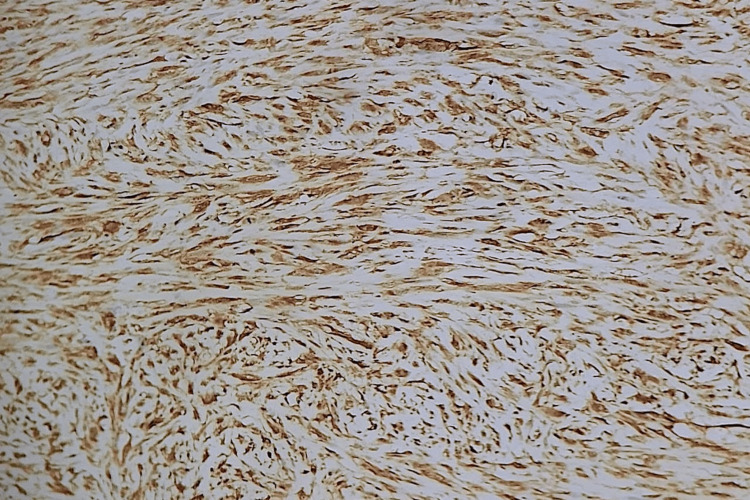
Immunohistochemical staining shows diffuse positivity for SMA SMA: smooth muscle actin

The patient benefited from a complete oncological excision of the tumor which was sent for histological study showing this time an intermediate grade leiomyosarcoma (grade II) of the National Federation of Cancer Centers (FNCLCC) with healthy resection margins. (Figures 3-6). Tumor resection began with a skin incision around the edge of the tumor. After careful hemostasis, we freed the tumor from its muscular cleavage plane while respecting the oncological resection margins. Skin closure was possible without the need for a skin graft.

The patient was referred to his treating oncologist for further treatment. His follow-up for eight months in orthopedic traumatology did not identify any post-operative complications, local recurrence, or secondary location.

## Discussion

Leiomyosarcoma is one of the most common types of soft tissue sarcoma. This tumor often manifests clinically as a painless mass, gradually increasing in size, adhering to surrounding structures, and well circumscribed. The tumor can sometimes ulcerate or fistulize into the skin. Pain and aesthetic or functional discomfort often motivate patients to consult [2].

There are no pathognomonic clinical or radiological signs of this tumor type; the diagnosis is therefore histological on biopsy or surgical resection specimen.

Histologically, leiomyosarcoma presents in the form of spindle cells of different sizes with abundant cytoplasm and elongated central nuclei. Multinucleated giant cells can be found. The immunohistochemical profile makes it possible to differentiate leiomyosarcoma from other malignant or benign tumors [3]. The risk of distant metastases is linked to the histological grade and size of the tumor. It is higher for large and high-grade lesions [4].

Leiomyosarcoma represents only 1% of adult and pediatric tumors [5]. To our knowledge, there is no case reported in the literature of a leiomyosarcoma next to the iliac crest, making our case exceptional. Murovic et al. reported a rare location of this tumor type on the lateral aspect of the left thigh invading the sciatic nerve [5]. Several clinical cases report localizations in the kidney, blood vessels, sphenoid sinus, and oral mucosa [6-9]. Camiade et al. reported a leiomyosarcoma at the expense of the iliac vein causing cruralgia [10].

The tumor location that our patient presents is considered extremely rare due to the lack of smooth muscle tissue in this area. There are no specific recommendations for the treatment of this tumor location. Generally speaking, oncological tumor resection is necessary in localized forms, coupled with chemotherapy or radiotherapy for forms with secondary locations [10,11]. Early diagnosis and adequate multidisciplinary management of this tumor considerably improve the prognosis.

## Conclusions

Leiomyosarcomas are relatively rare neoplasms that develop at the expense of smooth muscle fibers. The tumor exerts a tumor mass effect depending on its location. It can recur locally and metastasize distantly. Appropriate clinical and histological evaluation supplemented by immunohistochemistry is essential. Early diagnosis and multidisciplinary management improve the prognosis of these tumors. The treatment of this tumor is not yet codified, but only radical cancer surgery can achieve long-term survival.
